# Herpes Simplex Virus 1 Coinfection Modifies Adeno-associated Virus Genome End Recombination

**DOI:** 10.1128/JVI.00486-21

**Published:** 2021-06-10

**Authors:** Anita Felicitas Meier, Kurt Tobler, Kevin Michaelsen, Bernd Vogt, Els Henckaerts, Cornel Fraefel

**Affiliations:** a Institute of Virology, University of Zürich, Zurich, Switzerland; b Laboratory of Viral Cell Biology & Therapeutics, Department of Cellular and Molecular Medicine, KU Leuven, 3000 Leuven, Belgium; c Department of Microbiology, Immunology and Transplantation, KU Leuven, Leuven, Belgium; d Department of Infectious Diseases, School of Immunology and Microbial Sciences, King’s College London, London, United Kingdom; Cornell University

**Keywords:** AAV, genome end recombination, HSV-1, adeno-associated virus, herpes simplex virus type 1

## Abstract

Wild-type adeno-associated virus (AAV) can only replicate in the presence of helper factors, which can be provided by coinfecting helper viruses such as adenoviruses and herpesviruses. The AAV genome consists of a linear, single-stranded DNA (ssDNA), which is converted into different molecular structures within the host cell. Using high-throughput sequencing, we found that herpes simplex virus 1 (HSV-1) coinfection leads to a shift in the type of AAV genome end recombination. In particular, open-end inverted terminal repeat (ITR) recombination was enhanced, whereas open-closed ITR recombination was reduced in the presence of HSV-1. We demonstrate that the HSV-1 protein ICP8 plays an essential role in HSV-1-mediated interference with AAV genome end recombination, indicating that the previously described ICP8-driven mechanism of HSV-1 genome recombination may be underlying the observed changes. We also provide evidence that additional factors, such as products of true late genes, are involved. Although HSV-1 coinfection significantly changed the type of AAV genome end recombination, no significant change in the amount of circular AAV genomes was identified.

**IMPORTANCE** Adeno-associated virus (AAV)-mediated gene therapy represents one of the most promising approaches for the treatment of genetic diseases. Currently, various GMP-compatible production methods can be applied to manufacture clinical-grade vector, including methods that employ helper factors derived from herpes simplex virus 1 (HSV-1). Yet, to date, we do not fully understand how HSV-1 interacts with AAV. We observed that HSV-1 modulates AAV genome ends similarly to the genome recombination events observed during HSV-1 replication and postulate that further improvements of the HSV-1 production platform may enhance packaging of the recombinant AAV particles.

## INTRODUCTION

Adeno-associated virus (AAV) is a small nonpathogenic parvovirus which depends on helper factors to initiate and complete its replication cycle (1). Within the viral capsid, the AAV genome is present as a linear, single-stranded DNA (ssDNA) molecule of about 4.7 kb in length and is of either positive or negative polarity (2). The characteristic features of the AAV genome are the flanking 145-nucleotide-long inverted terminal repeat (ITR) sequences, which possess the ability to self-anneal into double hairpin structures (3). Within the host cell, the double hairpin structure of the ITR sequences serve as a 3′ OH primer for the single- to double-strand DNA conversion, leading to a duplex structure with one open and one covalently closed end (4–6). During lytic replication, this partially covalently closed linear structure is resolved by the large AAV Rep proteins Rep68/78, which possess a site-specific endonuclease activity (7). The Rep-mediated nicking at the so-called terminal resolution site (trs) results in the generation of another 3′ OH end, where the repair of the remaining sequence is initiated, leading to a linear double-stranded DNA (dsDNA) molecule with two open ends. Alternatively, these open-end dsDNA molecules are formed upon annealing of two individual incoming AAV genomes of opposite polarity.

In the absence of a helper virus, AAV has been shown to integrate its genome into the host cell chromosome (8–10). However, the predominant form of AAV2 DNA during latency appears to be extrachromosomal, circular, and double-stranded (11). The conversion to circular double-stranded episomes has also been reported for recombinant AAV genomes (12, 13) and has been shown to occur in the presence of a helper virus (14, 15).

While circularization is facilitated by the interaction of the AAV ITRs with DNA repair and recombination pathways (16–19), not much is known about the effect of the helper virus on the generation and processing of circular AAV DNA. A better understanding of these mechanisms would further our knowledge of AAV biology and the use of this virus as a vector for gene therapy. For example, if circular AAV DNA were not to serve as a substrate for the synthesis of packageable AAV DNA—preventing its formation, for example, through specific manipulations to the helper virus genome—AAV vector production might be improved.

Here, we investigated the effect of herpes simplex virus 1 (HSV-1) on the formation of the circular AAV2 genome structures. We hypothesized that HSV-1 has a direct impact on AAV genome end recombination since HSV-1 has the ability to manipulate cellular DNA repair pathways (20). In addition, it is known that the HSV-1 genome encodes proteins that facilitate recombination events (21, 22). Within the capsid, the 152-kb-long HSV-1 genome is present as a linear double-stranded DNA (dsDNA). Upon infection of a host cell, the HSV-1 genome recombines its unique long (*U_L_*) and unique short (*U_S_*) segments within the repeat regions, leading to genome inversions (23). The mechanism behind this event is unclear. Furthermore, the HSV-1 DNA binding protein ICP8 and the alkaline nuclease UL12 were shown to mediate DNA recombination (21, 22). The model for HSV-1-mediated recombination suggests that the alkaline nuclease UL12 removes nucleotides from the 5′ end of a dsDNA molecule, exposing a 3′ single-stranded tail. ICP8 then anneals the ssDNA tail to a complementary ssDNA sequence accompanied by a strand displacement of the complementary strand from the invading dsDNA molecule. It is important to note that only ssDNA and not dsDNA can serve as a complementary template. While the exonuclease activity of UL12 can be replaced by other exonucleases (even with 3′ to 5′ activity [22]), ICP8 appears to be indispensable for HSV-1-mediated recombination. Both ICP8 and UL12 were found to interact with multiple proteins from different cellular DNA damage response (DDR) pathways (24, 25). However, establishing their precise roles in the manipulation of DDR pathways has not been straightforward, as certain cellular responses have antiviral activities and are actively counteracted by HSV-1 (e.g., ICP0-mediated downregulation of DNA PKcs [26]), whereas other DDR proteins such as ATR are actively recruited to viral replication compartments and are essential for efficient viral replication (27, 28).

To study the direct impact of HSV-1 on the AAV genome structure and avoid the formation of structures arising during genome replication, we used replication-deficient AAV vectors, in particular, self-complementary AAV (scAAV) vectors, which self-anneal and thereby circumvent the necessity for double strand conversion. These vectors resemble the covalently closed duplex structure during a wild-type AAV infection, allowing us to specifically investigate the stages following double strand conversion.

## RESULTS

### HSV-1 interferes with GFP-expression from a recombination-dependent scAAV vector.

**FIG 1:**
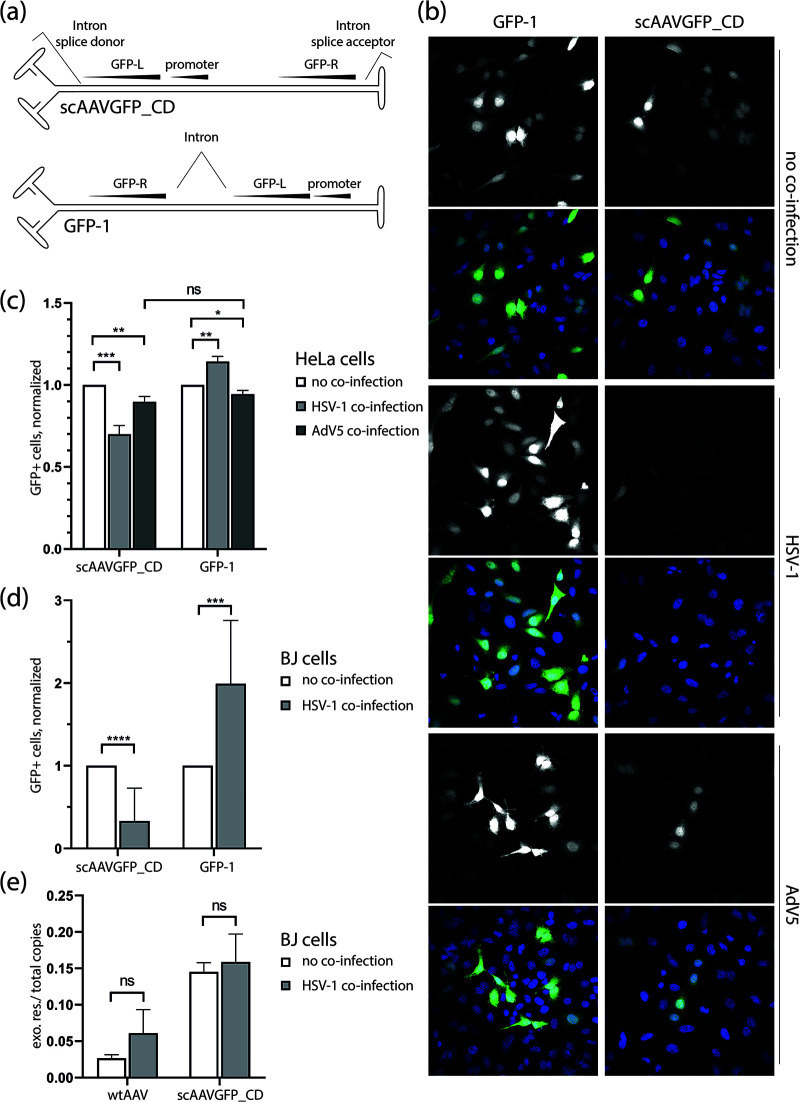
HSV-1, but not AdV5, interferes with scAAVGFP_CD GFP expression independent of circularization. (a) Schematic representation of scAAVGFP_CD and scAAV GFP-1 (GFP-1). (b) Representative photomicrographs of scAAV-infected HeLa cells (scAAVGFP_CD or scAAV GFP-1, gcp/cell = 1,000) in the presence or absence of a helper virus (HSV-1, PFU/cell = 1 or AdV5, PFU/cell = 2) at 23 hpi. The GFP signal in single-channel images is shown in white; in merged images, the GFP signal is shown in green together with DAPI (blue). (c) HeLa cells at 23 hpi; (d) BJ cells at 16 hpi; bar plot of flow cytometry data from scAAV-infected cells (scAAVGFP_CD or scAAV GFP-1, gcp/cell = 1,000) in the presence or absence of helper virus (HSV-1, PFU/cell = 1 or AdV5, PFU/cell = 2). The number of GFP+ cells was divided by the number of total cells to determine the ratio of GFP+ cells in each population. This ratio was then normalized to the ratio of GFP+ cells in non-coinfected samples. (e) Bar plot of qPCR data from exonuclease-resistant (circular) wtAAV or scAAVGFP_CD genome copies divided by the total genome copy number. BJ cells were infected with AAV (wtAAV or scAAVGFP_CD, gcp/cell = 1,000) in the presence or absence of HSV-1 (PFU/cell = 1) and analyzed at 20 hpi. (c to e) Data presented are means with standard deviation (SD) from at least 3 independent repeats with *P* values of helper virus coinfected samples compared to single-infected samples determined using the unpaired Student’s *t* test with equal SD. ****, *P* < 0.0001; ***, *P* ≤ 0.001; **, *P* ≤ 0.01; *, *P* ≤ 0.05; ns *P* > 0.05.

As a readout for AAV genome recombination, we made use of a self-complementary AAV vector (scAAVGFP_CD) in which the green fluorescent protein (GFP) sequence is split into two parts and placed at either end of the vector (18, 29) (Fig. 1a). To enable translation of a functional GFP, intron donor and acceptor sequences were placed adjacent to the ITR-repeat regions. Expression of a functional GFP is therefore only possible if the two ends come together, upon either intra- or intermolecular recombination (circularization or concatemerization). ScAAV GFP-1 was used as a control vector (Fig. 1a) (18). As for scAAVGFP_CD, the GFP sequence in scAAV GFP-1 contains an intron sequence. Transgene expression from scAAV GFP-1 depends on splicing but not on recombination of genome ends, therefore representing the ideal control vector for the assessment of genome end recombination. Self-complementary AAV (scAAV) vectors contain a truncated or defective ITR (dITR) sequence in the middle of the genome, which leads to the generation of recombinant viral genomes that self-anneal and are flanked by one open and one closed end. ScAAV vectors circumvent the need for single strand to double strand conversion before transcription and are ideal tools to study HSV-1-mediated genome manipulation following double strand conversion.

Infection with the scAAVGFP_CD or GFP-1 vector alone with 1,000 genome-containing particles/ cell (gcp/cell) resulted in a GFP-positive cell population within the range of about 10 to 25% in BJ cells and about 25 to 50% in HeLa cells in individual experiments. Transgene expression of the recombination-independent scAAV GFP-1 vector was enhanced upon HSV-1 coinfection (Fig. 1b to d). In contrast, HSV-1 coinfection led to a significant decrease of the number of GFP-positive cells in scAAVGFP_CD transduced cells (Fig. 1b to d). These results indicate that scAAV genome end recombination is impaired upon HSV-1 coinfection. AdV5 coinfection resulted in the overall reduction of GFP expression using both scAAV vectors but showed no significant difference between scAAVGFP_CD- and GFP-1-infected cells (Fig. 1b and c). Intramolecular genome end recombination of scAAVGFP_CD would lead to circularization of the genome and yield GFP-positive cells. Therefore, our first hypothesis was that HSV-1 inhibits circularization. To assess whether HSV-1 specifically reduces scAAV genome circularization or if the observed decrease of GFP-positive cells is due to other recombination events, we performed a quantitative PCR (qPCR) analysis on exonuclease V treated DNA isolated from AAV-infected cells in presence or absence of HSV-1. Exonuclease V specifically degrades linear ds- and ssDNA but preserves nicked and supercoiled circular DNA. No significant difference was found in the ratio of exonuclease-resistant (circular) to total genome copy numbers between coinfected cells and cells infected with scAAVGFP_CD or wild-type AAV2 alone (Fig. 1e). This shows that HSV-1 does not change the ratio of circular to total AAV genome copy numbers. Notably, however, the percentage of circularized genomes in wild-type AAV2-infected cells was approximately 5 times smaller than that of scAAVGFP_CD-infected cells.

### HSV-1 alters the type of scAAV genome end recombination.

**FIG 2:**
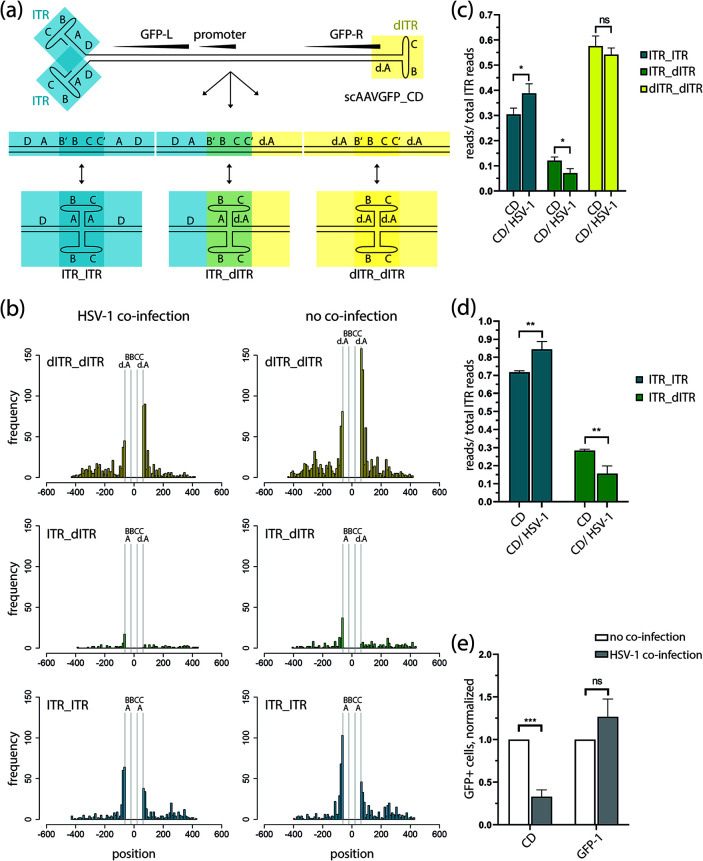
HSV-1 interferes with AAV genome end recombination. (a) Schematic representation of the different scAAVGFP_CD genome end recombination possibilities with the ITR motifs (d.A, A, B, C, and D) indicated. Wild-type AAV-derived ITR sequences are shown in blue boxes, and dITR sequences, in yellow boxes. (b) Histogram of the endpoint of all paired reads matching the three possibilities of end recombination shown in panel a with dITR_dITR in yellow, ITR_dITR in green, and ITR_ITR in blue. Position is relative to the ITR sequence, which is placed at 0 with the BBCC motif in the middle, flanked by the respective A motifs. The BBCC and A/d.A motifs are separated and indicated with gray vertical lines. The plotted paired Illumina sequencing data combines reads from triplicate experiments of BJ cells at 16 hpi; with scAAVGFP_CD-infected cells (gcp/cell = 20,000) in the presence or absence of HSV-1 (F-strain, PFU/cell = 1). (c) The number of reads (shown in panel b) with a specific ITR recombination type was divided by the number of total reads with recombined ITR, and the ratio was plotted. (d) Bar plot of the same data set shown in panel c; only reads containing ITR-dITR or ITR-ITR sequences were counted. The ratio of specific ITR recombination type to total number of reads of both ITR types was plotted. (e) Bar plot of flow cytometry data from scAAV-infected BJ cells (scAAVGFP_CD or scAAV GFP-1, gcp/cell = 20,000) in the presence or absence of HSV-1 (F-strain, PFU/-cell = 1). The number of GFP+ cells was divided by the number of total cells to determine the ratio of GFP+ cells in each population. This ratio was then normalized to the ratio of GFP+ cells in non-coinfected samples. Data presented are means with standard deviation (SD) from 3 independent experiments with *P* values of helper virus coinfected samples compared to single-infected samples determined using the unpaired Student’s *t* test with equal SD. ***, *P* ≤ 0.001; **, *P* ≤ 0.01; *, *P* ≤ 0.05; ns *P* > 0.05.

We used high-throughput sequencing to investigate the mechanism responsible for the observed HSV-1-induced reduction of scAAVGFP_CD-transduced cells. For this, we infected BJ cells with scAAVGFP_CD (gcp/cell = 20,000) in the presence or absence of HSV-1 (PFU/ cell = 1) and extracted extrachromosomal DNA (Hirt-extraction) at 16 h post-infection (hpi). Due to technical reasons, we had to increase the multiplicity of infection from 1,000 gcp/cell to 20,000 gcp/cell. However, despite the substantial increase in AAV particles per cell, HSV-1 continued to significantly decrease the number of GFP-positive cells (Fig. 2e). After extraction of extrachromosomal DNA, we sequenced the samples using the Illumina sequencing platform (paired 300-nucleotide-long reads). This approach enabled us to determine the quality and quantity of genome end recombinations. As a prerequisite of GFP-expression from the scAAVGFP_CD vector, the open genome end (wild-type ITR) has to recombine with the covalently closed, defective genome end (dITR), present in self-complementary AAV-vectors, resulting in a sequence that we designate here as ITR_dITR (Fig. 2a). Genome end recombination of two open ITR ends (ITR_ITR) or two dITRs (dITR_dITR) does not lead to GFP expression. We then analyzed the data by setting specific conditions that would reveal the quality of genome end recombination. Specifically, we selected pairs of reads that showed an alignment starting upstream of the common ITR sequences (before the common A sequence) and ending downstream of the common ITR sequence on the opposite side of the recombined ITR (Fig. 2b). With this approach we specifically selected read pairs long enough to (i) cover both sides of the common ITR sequences and (ii) identify the specific recombined ends. This approach revealed that the presence of HSV-1 resulted in a significant decrease of reads showing ITR_dITR recombination, whereas the number of reads containing wild-type ITR-end recombination (ITR_ITR) increased (Fig. 2c and d). Overall, reads containing dITR_dITR were the most abundant ITR recombination sequence, followed by ITR_ITR and then ITR_dITR. This is in accordance with previous findings from Choi et al. (29). Reads containing sequences resembling a dITR_dITR recombination were not significantly changed in the presence of HSV-1 (Fig. 2c). However, those dITR_dITR sequences are also present in scAAV viral stocks and therefore are not necessarily the result of a recombination event. With our approach, it is not possible to discriminate between dITR_dITR sequences resulting from actual end recombinations and the sequence found within the genome of scAAV vectors. Therefore, we removed the dITR_dITR sequence containing reads from our analysis, which resulted in the data shown in Fig. 2d. A clear change in ITR-dITR and ITR-ITR end recombinations was observed in the presence of HSV-1 (Fig. 2d). We therefore conclude that HSV-1 coinfection has an impact on the AAV genome conformation by altering the type of AAV genome end recombination. We further conclude that GFP expression from the scAAVGFP_CD rAAV vector is a valid readout to assess AAV genome end recombination.

In addition, we analyzed the frequency of genome end recombination, i.e., how many reads contained a sequence resulting from an ITR end recombination event in relation to the total AAV reads and found that HSV-1 coinfection led to a nonsignificant increase (data not shown). This further points at an HSV-1-mediated modification of the AAV end recombination mechanism.

We also found that in both the presence and absence of HSV-1, AAV genome end recombination with wild-type ITRs results in the removal of one B′-B-C-C′ motif. More precisely, the initial D-A-B′-B-C-C′-A sequence motif of each of the two genome ends is reduced to D-A-B′-B-C-C′-A-D upon recombination (Fig. 2a). Homologous recombination is therefore the most likely cellular pathway involved in genome end recombination. Comparable sequences of genome end recombination events were found for single-stranded recombinant AAV vectors (14). Similar to recombined wild-type ITRs, recombination of dITR sequences also leads to the deletion of one B′-B-C-C′ motif, resulting in d.A-B′-B-C-C′-d.A (dITR_dITR) or D-A-B′-B-C-C′-d.A when wild-type ITR sequences recombined with the dITR (ITR_dITR).

### ICP8 interferes with GFP expression from a recombination-dependent scAAV vector.

**FIG 3:**
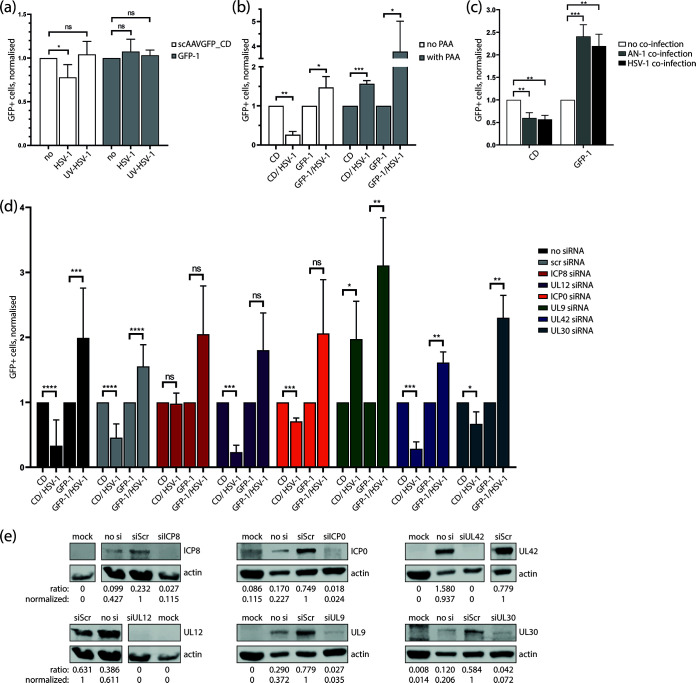
HSV-1 DNA replication factors interfere with scAAVGFP_CD GFP expression. (a) Bar plot of flow cytometry data from scAAV-infected BJ cells (scAAVGFP_CD or scAAV GFP-1, gcp/cell = 1,000) in the presence or absence of UV-treated (UV-HSV-1) or untreated HSV-1 (F-strain, PFU/cell = 1) at 16 hpi. (b) Bar plot of flow cytometry data from PAA-treated or PAA-untreated scAAV-infected BJ cells (scAAVGFP_CD or scAAV GFP-1, gcp/cell = 1,000) in the presence or absence of HSV-1 (F-strain, PFU/cell = 1) at 16 hpi. (c) Bar plot of flow cytometry data from scAAV-infected BJ cells (scAAVGFP_CD or scAAV GFP-1, gcp/cell = 1,000) in the presence or absence of the HSV-1 UL12-null mutant AN-1 or wild-type HSV-1 (KOS-strain) (PFU/cell = 1) at 16 hpi. (d) Bar plot of flow cytometry data from siRNA-transfected and scAAV-infected BJ cells (scAAVGFP_CD or scAAV GFP-1, gcp/cell = 1,000) in the presence or absence of HSV-1 (F-strain, PFU/cell = 1) at 16 hpi. In panels a to d, the number of GFP+ cells was divided by the number of total cells to determine the ratio of GFP+ cells in each population. This ratio was then normalized to the ratio of GFP+ cells in non-HSV-1-infected samples. Data presented are means with standard deviation (SD) from at least 3 independent experiments with *P* values of helper virus-coinfected samples compared to single-infected samples determined using the unpaired Student’s *t* test with equal SD. ****, *P* < 0.0001; ***, *P* ≤ 0.001; **, *P* ≤ 0.01; *, *P* ≤ 0.05; ns *P* > 0.05. (e) Western analysis of noninfected and nontransfected cells (mock), HSV-1 infected and nontransfected (no si), or HSV-1- and siRNA-transfected BJ cells (as indicated with siScr [scrambled siRNA]) harvested at 8 hpi (ICP8 and UL12 siRNA plus control samples) or 16 hpi (ICP0, UL9, UL42, and UL30 siRNA plus control samples) and stained with primary antibodies targeting ICP8, UL12, ICP0, UL9, UL42, UL30, and actin as indicated. The intensity of individual bands was quantified, and ratios were calculated by dividing the intensity value (area under the curve [AUC]) of the indicated band by the intensity value of the actin band. Normalization was done by dividing the indicated ratio by the ratio calculated for the scrambled siRNA (siScr)-transfected sample.

To investigate the HSV-1 factors required for the interference with AAV genome end recombination, we analyzed different aspects of the HSV-1 infectious cycle. First, we assessed whether HSV-1 gene expression is required using scAAVGFP_CD vector transduction efficiency as a readout. We found that coinfection with UV-treated HSV-1 did not reduce the transduction efficiency of either scAAVGFP_CD or scAAV GFP-1 (Fig. 3a), while coinfection with untreated HSV-1 reduced the transduction efficiency of scAAVGFP_CD (Fig. 1b to d and 3a) and enhanced transduction efficiency of scAAV GFP-1 (Fig. 1b to d) or had no significant effect (Fig. 3a). We therefore conclude that HSV-1 gene expression is indeed required for the interference with AAV genome end recombination. Although the overall effect of HSV-1 on the amount of GFP+ cells in scAAVGFP_CD- or scAAV GFP-1-infected cells remained the same throughout the study, we noticed a certain variation between experiments. Specifically, we experienced that the effect of HSV-1 was less pronounced in higher passages of BJ cells than in lower passages.

**FIG 4:**
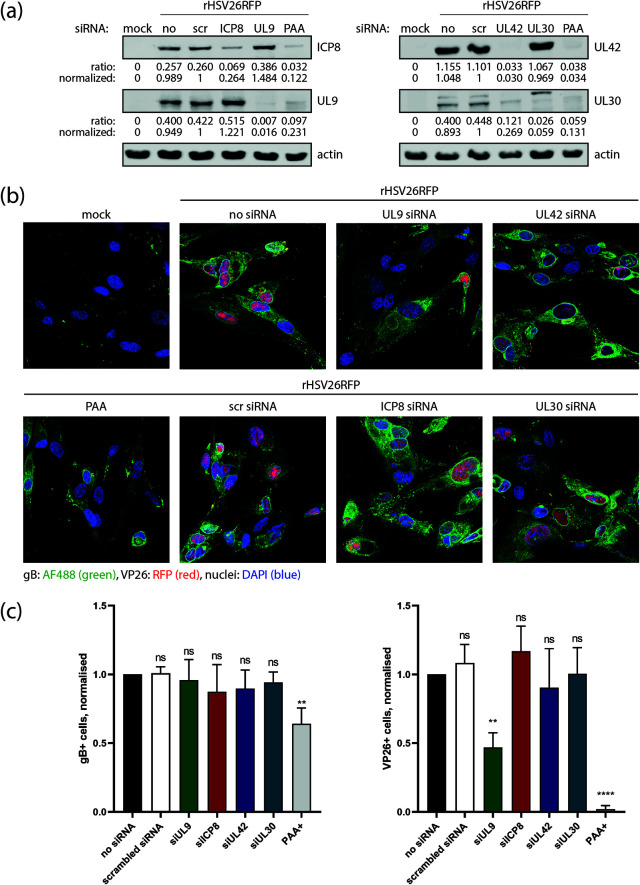
Impact of siRNA transfection and PAA treatment on HSV-1 leaky late and true late gene expression. BJ cells were transfected with siRNA targeting ICP8, UL9, UL42, or UL30. As control, cells were transfected with scrambled siRNA (scr), left untransfected (no and mock), or PAA-treated prior to infection. Cells were infected with rHSV26RFP (PFU/ cell = 1) or not infected (mock). (a) Western analysis of treated or untreated BJ cells harvested at 8 hpi and stained with primary antibodies targeting ICP8, UL9, actin, UL42, and UL30. The intensity of individual bands was quantified, and ratios were calculated by dividing the intensity value of the indicated band by the intensity value of the actin band. Normalization was done by dividing the indicated ratio by the ratio calculated for the scrambled siRNA (scr)-transfected sample. (b) Fixation and immunofluorescence staining of gB at 8 hpi. Photomicrographs were acquired using an inverted confocal laser scanning microscope. Nuclear staining is shown in blue (DAPI), gB in green, and RFP-conjugated VP26 in red. (c) Bar plot of data from computationally analyzed photomicrographs using CellProfiler. Approximately 100 nuclei were counted per sample and analyzed for gB or VP26 staining. The number of nuclei positive for either staining was normalized to the number of total nuclei and plotted. Data presented are means with standard deviation (SD) from at least 3 independent experiments with *P* values of siRNA- or PAA-treated samples compared to untreated (no siRNA) samples determined using the unpaired Student’s *t* test with equal SD. ****, *P* < 0.0001; ***, *P* ≤ 0.001; **, *P* ≤ 0.01; *, *P* ≤ 0.05; ns *P* > 0.05.

To identify the HSV-1 proteins responsible for the interference with AAV genome end recombination, we performed small interfering RNA (siRNA) knockdown experiments targeting immediate early and early HSV-1 genes and assessed the percentage of GFP+ cells upon scAAV infection. In addition, Western analysis was performed to confirm the knockdown upon siRNA treatment (Fig. 3e). Transfection of siRNA targeting ICP8 or UL9 showed a significant impact on the ability of HSV-1 to reduce the number of GFP+ cells in scAAVGFP_CD-infected fibroblasts (BJ) (Fig. 3d). While knockdown of ICP8 inhibited HSV-1 to lower the percentage of GFP+ cells, UL9 knockdown led to an increase of GFP+ cells in the presence of HSV-1. An increase of GFP+ cells was observed in scAAVGFP_CD as well as control cells infected with scAAV GFP-1, indicating that the presence of UL9 interferes with scAAV transduction. Knockdown of UL12 did not have an impact on the ability of HSV-1 to lower the number of GFP+ cells upon scAAVGFP_CD infection (Fig. 3d). This result was somewhat surprising, as UL12 in combination with ICP8 was found to mediate DNA ligation (21, 22). We therefore further assessed the role of UL12 by using the UL12-null mutant AN-1. AN-1 coinfection retained the ability to lower the percentage of GFP+ cells upon scAAVGFP_CD infection (Fig. 3c). We therefore conclude that UL12 is dispensable for the HSV-1-mediated interference with AAV genome recombination. Knockdown of ICP0 and the HSV-1 polymerase proteins UL30 and UL42 failed to revert the HSV-1-mediated interference with AAV genome end recombination (Fig. 3d). Phosphonoacetic acid (PAA), which is known to specifically inhibit HSV-1 replication (30), blocked the ability of HSV-1 to interfere with AAV genome end recombination (Fig. 3b). This result has two implications. First, the HSV-1 genome replication machinery might be required for HSV-1-mediated interference with AAV genome end recombination. Second, since expression of HSV-1 true late genes is dependent on HSV-1 genome replication, one or multiple true late gene products might be involved. Furthermore, ICP8 and UL9 are essential factors for HSV-1 genome replication (31), and therefore, their knockdown may also interfere with gene expression of HSV-1 true late genes. To assess this possibility, we examined the impact of ICP8 and UL9 siRNA-mediated knockdown on the expression of glycoprotein B (gB; a leaky late gene) and VP26 (a true late gene) (Fig. 4). Fibroblasts (BJ) at 24 h after siRNA transfection (or no transfection) were infected with an HSV-1 mutant expressing a red fluorescent protein (RFP)-tagged VP26 (rHSV26RFP) and, 8 h later, stained with a gB-specific antibody (Fig. 4b). Western analysis was performed in parallel to confirm the knockdown upon siRNA treatment (Fig. 4a). Computational analysis of the acquired photomicrographs revealed that PAA-treatment, which was used as a control, leads to a significant decrease of gB- and VP26-expressing cells (Fig. 4c). Furthermore, transfection of siRNA targeting ICP8, UL30, or UL42 did not lead to a significant decrease of gB- or VP26-expressing cells (Fig. 4c). Therefore, we conclude that siRNA-mediated knockdown of ICP8, UL30, and UL42, while clearly effective to reduce protein amounts (Fig. 3e and 4a), fails to interfere with the expression of true late genes. In contrast, transfection of siRNA targeting UL9 significantly reduced the expression of VP26 (Fig. 4b and c). While ICP8 knockdown did not affect the number of HSV-1 late gene-expressing cells, it inhibited the ability of HSV-1 to interfere with AAV genome end recombination. As shown in Fig. 3e and 4a, transfection of siRNA targeting ICP8 reduced protein levels to about 11.5% and 26.4%, respectively. Therefore, we conclude that reduction of ICP8 rather than a complete absence of it, is sufficient to interfere with the ability of HSV-1 to alter AAV genome end recombination. Thus, ICP8 is essential for HSV-1-mediated interference with AAV genome end recombination. In addition to ICP8, a true late gene might be involved, since PAA-treatment and UL9 knockdown inhibited both the interference of HSV-1 with AAV genome end recombination and HSV-1 true late gene expression. It is possible that, along with ICP8, UL9 is itself essential for the HSV-1-mediated interference of AAV genome end recombination. Nevertheless, because of its role in the expression of late genes, we cannot conclude this.

## DISCUSSION

Our data show that HSV-1 coinfection interferes with AAV genome end recombination. Specifically, we observed that coinfection with HSV-1 leads to differential genome end recombination structures and reduces the transduction efficiency of recombination-dependent scAAVGFP_CD vectors. In particular, open-end ITR recombination was enhanced, whereas open-closed ITR (ITR_dITR) recombination, leading to transduction, was reduced in the presence of HSV-1. We postulated that this could be attributed to the proposed mechanism for HSV-1-mediated recombination (21, 22), in which the viral proteins ICP8 and UL12 play an important role, although the role of UL12 can also be filled in by cellular nucleases (22). We found that the HSV-1 DNA binding protein ICP8 indeed plays an essential role in HSV-1-mediated interference with scAAVGFP_CD genome end recombination since the HSV-1-mediated reduction in transduction efficiency could not be observed upon knockdown of ICP8. Our data further show that UL12 is dispensable in the context of HSV-1-mediated AAV genome end recombination. According to the described model of HSV-1-mediated DNA recombination, the alkaline nuclease UL12 removes nucleotides from a dsDNA at the 5′ end, thus exposing a 3′ single-stranded tail (21). ICP8 then anneals the ssDNA tail to a complementary ssDNA sequence accompanied by a strand displacement of the complementary strand from the invading dsDNA molecule. It is important to emphasize that only ssDNA and not dsDNA can serve as a complementary template. This is consistent with the observation that in our studies, HSV-1 enhanced the recombination of AAV genome ITR ends, which are more likely to be present as ssDNA than the internal self-complementary ITR sequences (dITR). Following this line of thought, we hypothesize that upon self-annealing of the scAAV genome within the cell, the internal dITR forms a closed end, and the two flanking (wild-type) ITRs form a dsDNA end which is open at one side, thereby resembling the structure of a dsDNA break. The dsDNA (ITR) end will then get processed by cellular nucleases, exposing a single-stranded DNA tail. The HSV-1 ICP8 subsequently supports annealing and ligation of two exposed complementary ss ITR DNA tails. This leads to the observed enhanced recombination of flanking ITR ends. We can therefore conclude that HSV-1 directly modifies the structure of AAV genomes. Although we identified ICP8 as an essential factor during AAV genome end recombination interference, we also provide evidence that further factors such as products of the true late genes are involved.

As an alternative mechanism, we propose that ICP8 plays an essential role in the interference with AAV genome end recombination due to its interaction with cellular DNA damage response (DDR) pathways (24, 28). HSV-1 was found to recruit several factors (e.g., Mre11, Rad50, Nbs1) of the homologous recombination (HR) pathway into its replication compartments, suggesting an active role of these proteins in viral genome replication (20, 32, 33). DNA PKcs—a key protein of the nonhomologous end joining (NHEJ) pathway—on the other hand, was found to be targeted for degradation by the HSV-1 E3 ubiquitin ligase ICP0, thereby inhibiting downstream activation of the NHEJ pathway (26). Thus, targeted modulation of specific DDR pathways might be underlying the observed HSV-1-supported changes in AAV genome end recombination. This is supported by the fact that the sequences of the recombined AAV ITR ends found by us and others resemble structures expected to result from HR rather than NHEJ (14). HSV-1-mediated recruitment of proteins involved in HR to the replication compartments may further facilitate AAV genome end recombination.

Extrachromosomal circular dsDNA is the predominant structure during latent infection with wild-type AAV and recombinant AAV transduction, as seen in the context of gene therapy treatments, and can therefore be considered a relevant genome structure during AAV infections (11, 13). We know that circularization of scAAV vectors is facilitated by cellular DNA repair and recombination pathways (16–19). However, not much is known about the effect of coinfecting helper viruses on the generation and processing of circular AAV DNA or on the conversion of AAV genomes into other larger genomic structures such as concatemers. A better understanding of the impact of helper viruses on AAV genome processing mechanisms is pivotal to enhance our understanding of the production of AAV-based vectors for gene therapy. For example, if circular AAV DNA is not the preferred substrate for the synthesis of packageable AAV DNA, suppressing its formation might improve AAV vector production. The formation of circular genomes could be prevented by specific manipulation of the helper factors. For example, adenovirus expressing the E4 open reading frame 6 gene in the absence of E2a decreased the abundance of circular AAV DNA and favored the linear replication form monomer (Rfm) and dimer (Rfd) structures (34). Similarly, tweaking the helper functions of ICP8 might lead to a shift in suitable substrate for packaging. Although HSV-1 increased AAV genome circularization only slightly, the significant impact of HSV-1 on the AAV genome end recombination indicates that genome conformation and the transitioning into different replication intermediate structures might be of importance. How HSV-1 regulates the formation of various AAV genome replication intermediates will be the subject of further studies.

## MATERIALS AND METHODS

### Cells and viruses.

HeLa (cervical cancer, human), BJ (foreskin fibroblasts, human), 293T (embryonic kidney, human) and Vero (kidney, African green monkey) cells were obtained from ATCC (Manassas, VA, USA). All cell lines were maintained in normal cell culture medium (Dulbecco’s modified Eagle’s medium [DMEM] high glucose, supplemented with 10% fetal bovine serum [FBS], 2 mM glutamine, 100 units/ml penicillin, and 100 μg/ml streptomycin) in a humidified incubator at 37°C 95% air and 5% CO_2_.

Wild-type HSV-1 (strain F and strain KOS as the control strain for the experiment concerning AN-1) as well as rHSV26RFP were grown as described previously (35, 36). Briefly, a confluent layer of Vero cells was infected at a low multiplicity of infection (MOI) and harvested when cells showed complete cytopathic effect (CPE). Cells were scraped off the culture vessel and centrifuged, and the cell pellet was frozen and thawed three times. Supernatant and cell pellet were combined and centrifuged again. Aliquots were made from supernatant and stored at −80°C. PFU were determined by plaque assay. The HSV-1 UL12-null mutant AN-1 was kindly provided by S. Weller (University of Connecticut, USA). Cloning and production of AN-1 were described elsewhere (37).

Adenovirus type 5 was kindly provided by E. Henckaerts (KU Leuven, Belgium). Wild-type AAV2 was kindly provided by H. Büning (Hannover Medical School). Recombinant AAV (rAAV) was produced as described previously (38, 39). 293T cells were seeded and 24 h later (at about 70 to 80% confluence) polyethylenimine (PEI)-transfected with a plasmid containing the rAAV genome (for scAAVGFP_CD, the p-trs46D-GFP-CP; for scAAV GFP-1, the pGFP-1 plasmid) and pDG (40), a plasmid encoding adenovirus helper functions as well as AAV *rep* and *cap* genes. A total of 21 μg DNA and 73.6 μg PEI (Sigma-Aldrich) per 15-cm (diameter) round petri dish were added. Cells were harvested at 48 to 72 h posttransfection, and rAAV was purified using a fast protein liquid chromatography (FPLC) with AAV2-specific columns (Hi-TRAP AVB Sepharose HP, cytiva). Concentration of genome-containing particles (gcp) was determined by qPCR on DNase- and proteinase K-treated stock. Large quantities of rAAV stocks used for sequencing experiments were produced by the Viral Vector Facility, University of Zurich, Switzerland.

Plasmids used for the generation of scAAVGFP_CD (p-trs46D-GFP-CP) and scAAV GFP-1 (pGFP-1) stocks were kindly provided by D. McCarty (University of North Carolina at Chapel Hill).

### Infection protocol.

Cells were seeded 24 h before infection. The number of cells and specific dishes used are indicated in the sections below. Virus (rAAV or wtAAV in the presence or absence of HSV-1 or AdV5 or rHSV26_RFP) was diluted in an appropriate volume of DMEM (0% FBS). Low infection volumes were used to enhance infection while ensuring that the cell layer was completely covered. Cells were placed in a humidified incubator at 37°C and 5% CO_2_. Then, 1 to 2 h postinfection (hpi), supernatant was removed, and sufficient DMEM containing 2% FBS was added. Cells were incubated in a humidified incubator at 37°C and 5% CO_2_ for the indicated time.

### Sample preparation for microscopy.

HeLa cells were seeded at 80,000 cells/well in 24-well plates, and BJ cells were seeded at 90,000 cells/well in 12-well plates on glass slides. Then, 24 h later, cells were treated and incubated as indicated. Fixation was done after a 1× phosphate-buffered saline (PBS) wash using 3.7% formaldehyde in PBS (15 min, room temperature [RT]). Fixation was stopped with 0.1 M glycine in PBS for 5 min at RT. Cells were washed with PBS. For permeabilization, cells were treated with 0.2% Triton X-100 in PBS for 15 min at RT and immediately washed with PBS. Blocking was done using 3% bovine serum albumin (BSA) in PBS for 15 min at RT. The primary antibody was diluted in blocking medium (3% BSA in PBS) (mouse monoclonal anti-HSV-1 gB [H1817]; Novus Biologicals, Littleton, CO; 1:200) and incubated for 1 h at RT. Cells were washed 3 times with PBS for 15 min. The secondary antibody was diluted in blocking medium (Alexa Fluor 488 goat anti-mouse IgG; Invitrogen; 1:1,000) and incubated for 1 h at RT. Cells were washed 3 times with PBS for 15 min. Cover slides were mounted after washing with deionized H_2_O in mounting medium containing DAPI (4′,6-diamidino-2-phenylindole) (ProLong diamond antifade mountant with DAPI; Invitrogen).

### Microscopy sample data analysis.

Photomicrographs were acquired using a Leica inverse SP8 confocal laser scanning microscope (CLSM) using the appropriate lasers and imaging channels. Images were acquired at a 63× magnification. For quantification, images were analyzed using the open-source software CellProfiler (41). Using the image analysis software, nuclei were identified (based on DAPI staining) and discriminated for VP26 and gB staining. Data were then further plotted and analyzed using Prism (see below).

### Flow cytometry.

For flow cytometry analysis, BJ cells were seeded 24 h before infection at 90,000 cells/well in 12-well plates. HeLa cells were seeded in 24-well plates at 80,000 cells/well. Cells were harvested for flow cytometry analysis at the indicated time points as follows. First, cells were washed with PBS and detached using 0.05% Trypsin-EDTA and transferred to a fluorescence-activated cell sorter (FACS) tube with complete medium. Cells were pelleted and supernatant was discarded. The cell pellet was resuspended in a PBS solution supplemented with 10% FBS and analyzed using a CytoFLEX S flow cytometer (Beckman Coulter).

### Circularization assay using exonuclease.

BJ cells were seeded (250,000 cells/well in 6-well plates) and 24 h later infected (AAV, gcp/cell = 1,000; HSV-1, PFU/cell = 1). At the indicated time point, cells were washed with 1× PBS and detached using 0.05% Trypsin-EDTA. After centrifugation, the supernatant was discarded, and the cell pellet was resuspended in 100 μl proteinase K solution (10 μg/ml proteinase K, 0.1% SDS in 1× New England Biolabs [NEB] buffer 4 solution) and incubated at 55°C for 1 h. Proteinase K was inactivated at 95°C for 15 min. Samples were prediluted 1:10 before exonuclease treatment. Then, 3 μl of prediluted sample was added to 27 μl exonuclease solution (10 U of exonuclease V [RecBCD; NEB], 1 mM ATP in 1× NEB buffer 4 solution) for exonuclease-treated samples or in 27 μl 1× NEB buffer 4 solution for control samples and incubated for 30 min at 37°C. Samples were then heat-inactivated at 70°C for 30 min. The concentration of genome copy numbers was determined using qPCR.

### qPCR protocol and primers.

A 2.5-μl sample was added to 7.5 μl reaction mix (containing SYBR green master mix and primers), and qPCR was performed using a QuantStudio 3 real-time PCR system (Thermo Fisher). The final reaction mix consisted of 0.2 μM forward and 0.2 μM reverse primer in 1× SYBR green master mix (PowerUp SYBR green master mix or SYBR green PCR master mix; Applied Biosystems). For quantification of scAAVGFP_CD and scAAV GFP-1-containing samples, primers that bind to sequences within the cytomegalovirus (CMV) promoter were used. For quantification of wtAAV-containing samples, *rep* primers were used (Table 1). All primers were synthesized by Microsynth, Switzerland, and are listed in Table 1.

**TABLE 1:** Primer list

Location	Forward	Reverse
CMV	5′-ATGACCTTATGGGACTTTCCTACTTGG	5′-CCCGTGAGTCAAACCGCTATCC
*rep*	5′-ATTGACGGGAACTCAACGAC	5′-ATTCATGCTCCACCTCAACC

### UV treatment of HSV-1.

HSV-1 virus stock was added to a 24-well plate and UV-treated for 10 min on ice (254 nm, UVC500 UV Crosslinker; Hoefer). UV-treated HSV-1 was used immediately for infection. Infectivity was assessed using fluorescence microscopy (immunofluorescence staining for VP16 and ICP4; data not shown). Particle integrity was assessed using negative staining electron microscopy. A duration of UV treatment which did not interfere with infection but disabled gene expression was chosen.

### PAA treatment.

Phosphonoacetic acid (PAA; Sigma-Aldrich) was added to cell supernatant at a final concentration of 400 μg/ml 2 to 0.5 h before infection and maintained throughout the experiment until cells were harvested.

### siRNA transfection.

The siRNA transfection was performed according to the manufacturer’s protocol. Briefly, Lipofectamine RNAi MAX reagent (Invitrogen) was added to Opti-MEM (Gibco), incubated for 5 min at RT, and added to 12-well plates. siRNA pools containing 3 different siRNAs (Table 2) (or scrambled control siRNA-A sc-37007, SCBT) targeting the same gene were added to the Lipofectamine mixture and incubated for 20 min at RT. Then, 40 pmol siRNA and 3 μl lipofectamine were added per well. BJ cells were added to the siRNA-Lipofectamine mixture (90,000 cells/well) and incubated for 24 h before infection. Sequences of the used siRNA are listed in Table 2.

**TABLE 2:** HSV-1-specific siRNAs

Target gene	siRNA 1	siRNA 2	siRNA 3	Manufacturer
*ICP0*	5′-GCA CCA UCC CGA UCG UGA ATT-3′	5′-GCA CGG ACA CGG AAC UGU UTT-3′	5′-CCG ACA GUC UGG UCG CAU UTT-3′	Thermo Fisher
*ICP8*	5′-UCU GGU UUG CCA UGU AGU ATT-3′	5′-UAA AGU UGA UCG CGC CGU ATT-3′	5′-UGA ACG CGA UGG UCU CGA UTT-3′	Microsynth, Switzerland
*UL30*	5′-GUA CUA UAG CGA AUG CGA UTT-3′	5′-AGA UAA AGG UGA ACG GCA UTT-3′	5′-GAC GUG UAC UAC UAC GAG ATT-3′	Thermo Fisher
*UL42*	5′-CCG UUA CGC GUG UUA CUU UTT-3′	5′-CCA UCC CGG GUU AAU GUA ATT-3′	5′-CUG GAA CAC UCG CAA UUC ATT-3′	Thermo Fisher
*UL9*	5′-GCC UUA UCU UGG ACG CGU UTT-3′	5′-CCA AUU ACA UUA UGA ACG ATT-3′	5′-ACA AAU UCC GUU ACA AAC ATT-3′	Thermo Fisher

### Western blot analysis.

BJ cells were seeded at 90,000 cells/well in 12-well plates and infected 24 h later as indicated. Total cell lysates were harvested 8 hpi. Whole-cell lysates were separated on 10% sodium dodecyl sulfate (SDS)-polyacrylamide gels, transferred to nitrocellulose membranes, probed with primary antibodies, and stained using anti-mouse IgG antibodies (IRDye 680RD goat anti-mouse or IRDye 800CW goat anti-mouse antibody; LI-COR Biosciences; 1:10,000). As primary antibodies, the following reagents were used: anti-UL9 (mAb13925, ascites, obtained from N. Stow, 1:500), anti-UL42 (mouse Z1F11, obtained from R. Everett, 1:1,000), anti-UL30 (mAb13429, ascites, obtained from N. Stow, 1:500), anti-ICP8 (MAb, ab20193; abcam; 1:1,000), and anti-actin (Sigma-Aldrich; 1:10,000). For antibody stripping, membranes were incubated for 15 min with stripping buffer (Thermo Scientific) and washed three times with phosphate-buffered saline (PBS). Images were acquired using the LI-COR imaging system Odyssey Fc (LI-COR Biosciences). The intensities of individual bands were quantified by measuring the area under the curve (AUC) using Fiji (42).

### Hirt extraction.

Extraction of extrachromosomal DNA was performed according to the Hirt protocol (43). Briefly, cells were washed with PBS and detached using 0.05% trypsin-EDTA. The cell pellet was resuspended in 50 μl Tris-buffered saline (TBS) (50 mM Tris-HCl, 150 mM NaCl, pH 7.5), and 500 μl Hirt buffer (0.6% SDS, 10 mM Tris-HCl, 10 mM EDTA, pH 7.5) was added and incubated at room temperature for 1 h. After the addition of 120 μl of a 5 M NaCl solution, the sample was incubated at 4°C for at least 12 h. For phenol/chloroform extraction of DNA, the sample was centrifuged for 10 min at 15,500 × *g* and 4°C. The supernatant was transferred into a fresh tube, and 1 volume of phenol:chloroform:isoamyl alcohol (25:24:1, vol/vol) was added. The sample was centrifuged for 5 min at 15,500 × *g* at 4°C. The supernatant was transferred into a fresh tube, and 1 volume of chloroform was added. The sample was centrifuged for 1 min at 15,500 × *g* at 4°C. The supernatant was transferred into a fresh tube, and 2.5 volumes of EtOH (pure) and 0.1 volume 3 M NaAc pH 5.5 were added, and the mixture was incubated at −80°C for at least 20 min to precipitate DNA. The sample was centrifuged for 10 min with 18,000 × *g* at 4°C, and the supernatant was discarded. The pellet was washed with 70% EtOH. After centrifugation for 10 min at 18,000 × *g* and 4°C, the supernatant was removed, and the pellet was air-dried before being resuspended in 10 mM Tris-HCl, pH 8.5.

### Illumina-Seq: sample preparation and data analysis.

BJ cells were seeded into one tissue culture petri dish (10 cm in diameter [Nunc], 9 × 10^5^ cells/dish) per condition 24 h prior to infection. Cells were infected as described above and harvested for Hirt extraction at 16 hpi. Then, 1 ng DNA of each sample was used to prepare the DNA library and for indexing according to the manufacturer’s protocol (Nextera XT DNA library prep kit, FC-131-1096, Nextera XT index kit v2 SetA, FC-131-2001; Illumina, San Diego, CA, USA). Sequencing was performed with an Illumina MiSeq instrument using the MiSeq reagent kit v3 (MS-102-3003; Illumina, San Diego, CA, USA). Paired reads (300 nucleotides [nt] in length) were mapped with Bowtie 2 (44) against the three combinations of ITR_ITR, ITR_dITR, and dITR_dITR. A total of 1,000 nt flank the various ITRs on both sides. Pairs of reads were combined on one line with BEDTools (45). The resulting data frame was opened in R (https://www.R-project.org). Read pairs spanning the entire ITR combinations were selected and counted for each combination.

### Data analysis.

SP8 CLSM images were analyzed using CellProfiler (41). First, nuclei were identified from blue-channel (DAPI) images. VP26 and gB signals were identified on red and green channel images, respectively. Correlation of previously defined nuclei with either VP26 or gB signal yielded the number of positive nuclei.

All bar plots were generated using GraphPad Prism software v8 for Microsoft Windows. *P* values (unpaired Student’s *t* test with equal standard deviations [SD]) were calculated using GraphPad Prism software v8 for Microsoft Windows.
